# Vasopressin versus epinephrine during cardiopulmonary resuscitation of asphyxiated newborns: A study protocol for a prospective, cluster, open label, single-center, randomized controlled phase 2 trial – The VERSE-Trial

**DOI:** 10.1016/j.resplu.2023.100459

**Published:** 2023-08-31

**Authors:** M. Ramsie, P.-Y. Cheung, B. Law, G.M. Schmölzer

**Affiliations:** a Centre for the Studies of Asphyxia and Resuscitation, Neonatal Research Unit, Royal Alexandra Hospital, Edmonton, Alberta, Canada; b Department of Pediatrics, University of Alberta, Edmonton, Alberta, Canada

**Keywords:** Infants, Newborn, Neonatal resuscitation, CHEST compressions, Asphyxia, Epinephrine, Vasopressin, Adrenaline

## Abstract

**Introduction:**

Current neonatal resuscitation guidelines recommend the use of epinephrine during neonatal cardiopulmonary resuscitation (CPR). However, newborns receiving epinephrine continue to have high rates of mortality and neurodevelopmental disability. The infrequent need for neonatal CPR, coupled with an inability to consistently anticipate which newborn infants are at risk of requiring CPR, explains the lack of high-quality evidence (i.e., large randomized clinical trials) to better guide healthcare providers in their resuscitative effort. Therefore, we need neonatal data to determine the optimal vasopressor therapy during neonatal CPR. The current pilot trial will examine the efficacy of vasopressin versus epinephrine during CPR of asphyxiated newborn infants.

**Methods and analysis:**

The trial will be a prospective, cluster, open label, single-center, randomized controlled trial on two alternative cardiovascular supportive medications. This study will assess the primary outcome of time to return of spontaneous circulation (ROSC) in newborns requiring CPR in the delivery room who were treated with either vasopressin (intervention) or epinephrine (control). Secondary outcomes such as infant mortality and other clinical outcome measures will also be collected. An estimated 20 newborns will be recruited, and comparisons will be made between asphyxiated infants treated with either drugs.

**Ethics and dissemination:**

This study has been approved by the Research Ethics Board at the University of Alberta (June 16, 2023). Study findings will be published in peer-reviewed journals, presented at conferences, and communicated to relevant participants and stakeholders.

**Trial registration:** ClinicalTrial.gov Identifier: NCT05738148. Registered February 21, 2023.

## Introduction

### Background and rationale {6a}

Current neonatal resuscitation guidelines recommend epinephrine during cardiopulmonary resuscitation (CPR) at a dose of 0.02 mg/kg, preferably given intravenously (i.v.), with repeated doses every 3 min until return of spontaneous circulation (ROSC).^1^, ^2^ These recommendations are based on adult studies and neonatal animal data, as neonatal human data are limited.^3^, ^4^, ^5^, ^6^, ^7^ Epinephrine, an endogenous catecholamine, which results in systemic and coronary vasoconstriction, improved cardiac function as well as increases myocardial oxygen demand, respiratory and metabolic acidosis.^8^ Although epinephrine has been used for decades during neonatal CPR, the optimal timing, dose, and route are unknown.^3^, ^4^, ^5^

Alternatively, vasopressin, an antidiuretic hormone with vasoactive action through V1 receptor activation, might be beneficial due to its postulated effects including combined pulmonary vasodilation and systemic vasoconstriction, not affected by respiratory and metabolic acidosis, and no increase in myocardial oxygen demand.^4^, ^9^ Large adult trials reported that, compared with epinephrine, vasopressin was associated with significantly higher rates of hospital admission (29% vs. 20% *p* = 0.02) and hospital discharge (5% vs. 2%, *p* = 0.04) when asystole was the cause of cardiac arrest.^10^, ^11^ As asphyxia leading to asystole is the main cause for cardiac arrest in newborns, vasopressin might be beneficial during neonatal CPR.^1^, ^2^, ^12^, ^13^, ^14^

To date, only 3 neonatal animal studies compared vasopressin with epinephrine. One used a transitional near-term-sheep model to compare vasopressin (0.4U/kg) and epinephrine (0.03 mg/kg) and reported time to ROSC [13 ± 6 min vs. 8 ± 2 min, no *p*-value reported] and survival rates [3/9 vs. 7/10, *p* = 0.179] between vasopressin and epinephrine.^15^ Another used a post-transitional cardiac arrest piglet model to compare high and low dose vasopressin (0.2 and 0.4U/kg) and epinephrine (0.01 and 0.03 mg/kg). This study did not report time to ROSC but reported a significantly higher survival rate with vasopressin (0.4U/kg) vs. low-dose epinephrine (0.01 mg/kg) [9/10 vs. 5/13 (*p* < 0.05)], but no significant difference in survival when compared to high-dose epinephrine (0.03 mg/kg, 6/11 survival).^16^ Most recently, our group compared vasopressin (0.4U/kg) and epinephrine (0.02 mg/kg) in a similar piglet model of post-transitional cardiac arrest and observed similar median (IQR) time to ROSC with 106 (93–140) sec with vasopressin vs 128 (100–198)sec with epinephrine (*p* = 0.28) with significantly longer post-resuscitation survival time with vasopressin with 240 (240–240) min compared to epinephrine 65 (30–240) min (*p* = 0.02).^17^

Despite being used for decades during neonatal CPR, there is little information on the efficacy of using epinephrine during neonatal CPR^3^, ^4^, ^5^ and clinical trials are needed to examine the optimal vasopressor therapy during neonatal CPR. In the current trial, we aim to compare the efficacy of vasopressin and epinephrine during neonatal CPR. We hypothesize that in newborns who require CPR, vasopressin compared to epinephrine will reduce time to achieve return of spontaneous circulation, defined as a heart rate of > 60/min for 60sec.

### Objectives {7}

The primary objective is to compare vasopressin and epinephrine during neonatal CPR to assess if vasopressin improves time to ROSC in preterm and term newborns.

### Trial Design {8}

**Fig. 1:**
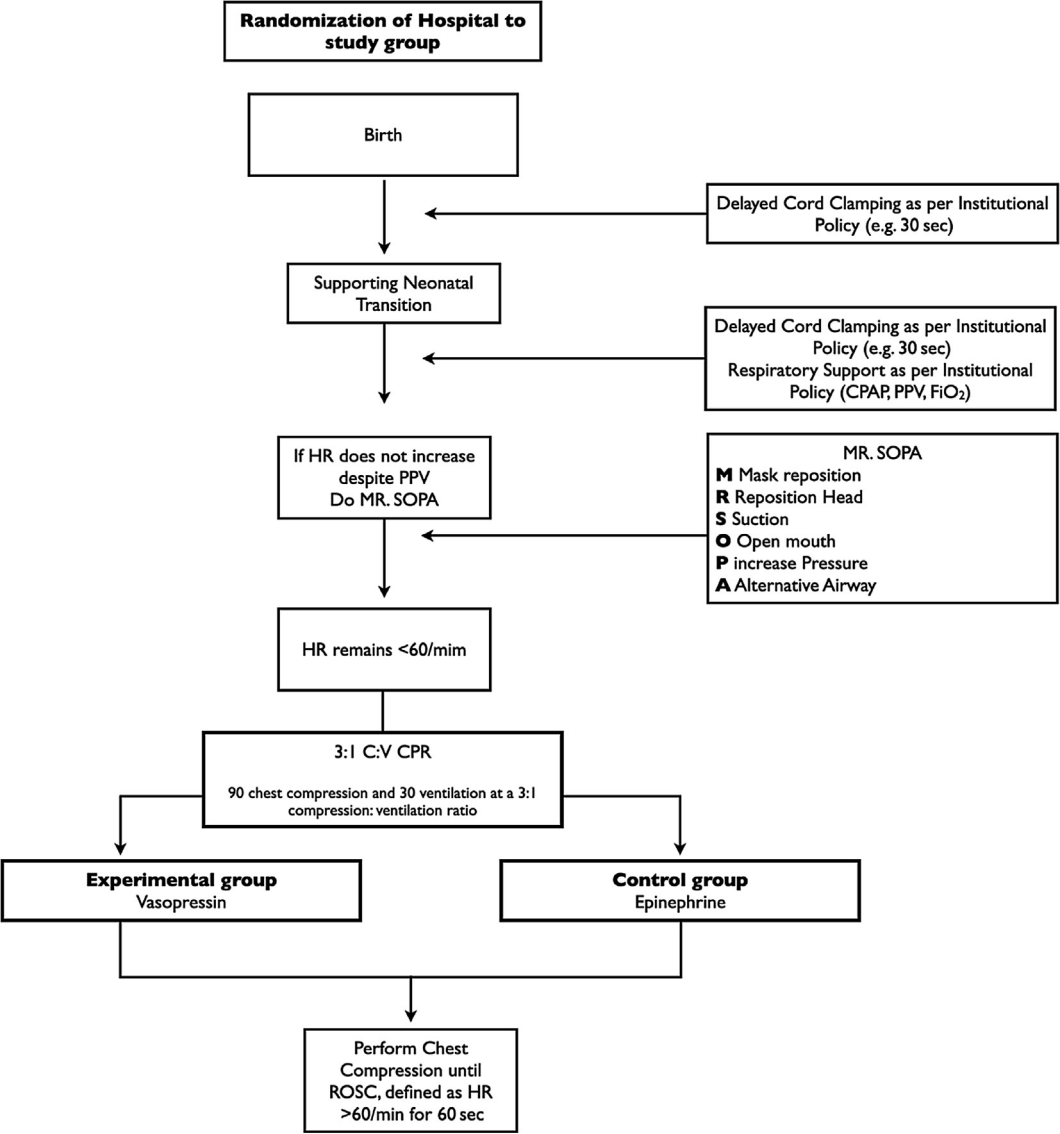
Trial flow chart.

Prospective, open label, cluster, single-center, pilot randomized controlled trial (RCT) comparing two cardiovascular supportive medications during neonatal CPR (trial design is presented in Fig. 1). The trial approach will be using a deferred consent approach.^18^

## Methods and analysis

### Study setting {9}

This study will be conducted in the delivery room at the Royal Alexandra Hospital, Edmonton, Alberta, Canada, a tertiary center for high-risk obstetrics, perinatal and neonatal medicine.

### Eligibility criteria {10}

Term and preterm infants who require CPR in the delivery room are eligible to receive the intervention.

Infants are excluded if they have a congenital abnormality or condition that might have an adverse effect on breathing or ventilation (e.g., congenital diaphragmatic hernia), or congenital heart disease requiring intervention. Infants are also excluded if their parents decline to give consent after birth for data collection.

### Who will take informed consent? {26a}

We will obtain individual consent after birth for data inclusion in the trial as per Tri-Council Policy Statement in Human Research guidelines for research in “Individual Medical Emergencies”^19^. In cases where the patient does not achieve ROSC, consent will be sought during bereavement.

## Interventions

### Explanation for choice of comparators {6b}

The two comparators are vasopressin (intervention group) and epinephrine (control group) during neonatal CPR. Epinephrine was chosen as control, as it is the current standard of care per international resuscitation guidelines despite limited evidence from clinical trials. Vasopressin was chosen due to its pharmacological properties, data demonstrating equivalence or superiority to epinephrine in adult CPR trials, and promising data from neonatal animal models.

### Intervention description {11a}

#### General interventions

The initial steps of the resuscitation, cord management, and mask ventilation will be according to the current neonatal resuscitation guidelines.^1^, ^2^ Chest compressions (CC) are started if heart rate remains < 60/min despite effective ventilation. CC are performed using the 2-thumb encircling technique, a 3:1 compression:ventilation ratio (90 chest compression and 30 ventilations per minute), 100% oxygen, and a CC depth of 1/3 anterior-posterior chest diameter.^1^, ^2^

#### “Epinephrine group”

Epinephrine will be administered either via umbilical vein catheter (0.02 mg/kg per dose) or via endotracheal tube (0.1 mg/kg) every three to five minutes as needed.^1^, ^2^ CC and epinephrine will be continued until ROSC.

#### “Vasopressin group”

Vasopressin will be administered either via umbilical vein catheter (0.4 IU/kg per dose)^17^ or via an endotracheal tube (8 IU/kg) every three to five minutes. After a maximum of two doses of vasopressin (if there is no ROSC), the clinical team must convert to administer epinephrine (0.02 mg/kg per dose) until ROSC as a reversion to standard of care.

#### Treatment period

Until ROSC, cessation of resuscitation efforts, or up to a maximum of 60 minutes.

#### Determination of ROSC

ROSC will be defined as an increase in heart rate > 60/min by ECG monitoring and auscultation, which is maintained for 60sec. Pulseless electrical activity will not be considered as ROSC.^12^, ^13^, ^14^, ^20^, ^21^

### Criteria for discontinuing or modifying allocated interventions {11b}

The attending clinician can withdraw the participant from the trial and revert to the standard treatment (epinephrine) at any time during CPR. The reasons shall be documented. There are no pre-specified criteria for discontinuation of participants from the trial. The discontinuation of participants in the trial will not result in replacement with new participants.

### Strategies to improve adherence to intervention protocols {11c}

The chief investigator consents to data evaluation being performed by the person in charge of monitoring in accordance with the monitoring plan, to ensure satisfactory data collection and adherence to the study protocol. The tasks of the investigator include maintenance of these patients' medical files as comprehensively as possible; this includes information concerning medical history, accompanying diseases, inclusion in the trial, data about visits, results of investigations, dispensing of medication, and adverse events. The monitor will also be permitted to perform data evaluation and draw comparisons with the relevant medical files in accordance with the standard operating procedures and Good Clinical Practices as described by the International Conference on Harmonization guidelines at predetermined intervals, to ensure adherence to the study protocol and continuous registration of data.

### Relevant concomitant care permitted or prohibited during the trial {11d}

All medications are permitted before and during the trial. Epinephrine can be given as a rescue treatment during ongoing CPR and will be given after two doses of vasopressin if there is ongoing CPR. All standard medications and care will be administered as per institutional practices after the ROSC including the administration of epinephrine infusion for inotropic effects.

### Provisions for post-trial care {30}

Post-trial care will be according to local hospital policy.

### Outcomes {12}

Outcomes will be reported as per Recommended Guideline for Uniform Reporting of Neonatal Resuscitation using the Neonatal Utstein.^22^

The primary outcome will be time to ROSC, defined as an increase in heart rate > 60/min, which is maintained for 60 sec.

Secondary outcomes among others will include neonatal mortality (Neonatal death < 28 days) and morbidities: Types and timing of brain injuries (reported either via magnetic resonance imaging (MRI) or head ultrasound), delivery room interventions (including mask ventilation, intubation, chest compression, use of vasopressor), admission temperature, therapeutic cooling, mechanical ventilation, use of vasoactive substances, acute kidney injury (24 hours serum creatinine, cumulative urine output) and blood gases and serum sodium levels at 8–12 and 24–28 hours, infection/sepsis, necrotizing enterocolitis, bronchopulmonary dysplasia, retinopathy of prematurity. NIRS will be applied for the continuous monitoring of cerebral regional oxygen saturation for 72 hours. Doppler study of cranial ultrasounds and Targeted Neonatal Echocardiography to assess cerebral blood flow and cardiac function, respectively, will be performed in the first 24 hours as per clinical indication.

### Participant timeline {13}

Following birth, infants (term or preterm) requiring CPR in the delivery room will receive either epinephrine or vasopressin (study site randomization of vasopressor is described in ‘Sequence generation {16a}’) and ‘Intervention description {11a}.’

### Sample size {14}

This will be the first trial to compare two medications during neonatal CPR in the delivery room. Therefore, no sample size calculation has been performed. The trial aims to recruit all infants requiring epinephrine or vasopressin in the delivery room with the 2-year time frame. In 2022, between 5 and 10 infants required CPR at birth, which we expect will be similar during the trial.

### Recruitment {15}

Recruitment is estimated to begin October 1, 2023, with a predicted primary completion date (final data collection for primary outcome measures) of September 31, 2025.

## Assignment of interventions: Allocation

### Sequence generation {16a}

At the beginning of the trial, the Royal Alexandra site will be randomized to either start with Vasopressin group (“intervention group”) or Epinephrine group (“control group”) for the first year. For the second year, the intervention group will be changed to the other group.

### Concealment mechanism {16b}

Not applicable.

### Implementation {16c}

Not applicable.

## Assignment of interventions: Blinding

### Who will be blinded {17a}

There will be no blinding during the recruitment phase as each year is allocated to one treatment arm and therefore the clinical team nor the outcome assessor (primary outcome is ROSC) cannot be blinded to the group assignment. The trial statistician will be blinded to the group allocation during statistical analysis.

### Procedure for unblinding if necessary {17b}

Clinicians at the resuscitation and involved with post-resuscitative care will be aware of the vasopressor received, therefore unblinding is not necessary.

## Data collection and management

### Plans for assessment and collection of outcomes {18a}

Infants will be recruited over a period of 24 months. All data will be collected on an electronic Case Report Form (eCRF) and entered into REDCap.

Incidence and causes of mortality will be recorded. Cerebral injury will be assessed with either cranial ultrasound or MRI brain if performed prior to discharge. If an infant die prior to any neuro-imaging, the study team will examine reports from any post-mortem examination, such as imaging or autopsy after parental consent. Autopsy could include imaging alone or full autopsy. Information on morbidity will be collected through case history until discharge. Secondary outcomes will be collected with case history.

### Plans to promote participant retention and complete follow-up {18b}

All participants are admitted to the neonatal intensive care unit, which will allow retention of participants until discharge, which is the end of study follow-up.

### Data management {19}

Source data will be registered in the participant’s medical records into the eCRF. Data will be stored for statistical analysis at the Biostatistics Unit, Women and Children's Health Research Institute, University of Alberta, Edmonton, Canada.

### Confidentiality {27}

Information from the trial will be held in strict confidentiality, and any published results will be completely anonymous. After the end of trial, the data will be archived for 15 years according to good clinical practice guidelines. At each trial-site the data flow will be monitored according to the Good Clinical Practices principles by a locally appointed external monitoring committee.^23^

### Plans for collection, laboratory evaluation, and storage of biological specimens for genetic or molecular analysis in this trial/future use {33}

No biological specimens will be collected or stored.

## Statistical methods

### Statistical methods for primary and secondary outcomes {20a}

The primary outcome is time to ROSC. The data will be analyzed on an intention-to treat basis and will include all randomized participants. A per protocol analysis will also be conducted using the data from the actual allocation of participating infants. A survival analysis will be used to analyze the difference in time to ROSC between intervention and control groups. To account for cluster randomization, Cox proportional hazards regression with time to ROSC as an outcome and allocation group as an independent variable will be created.

Secondary outcomes will be compared using Student’s *t*-test for parametric and Mann-Whitney *U*-test for nonparametric comparisons of continuous variables, and χ^2^ for categorical variables. All data will be presented as mean (standard deviation, SD) for normally distributed continuous variables and median (interquartile range, IQR) when the distribution is skewed. All *p*-values will be 2-sided and *p* < 0.05 will be considered statistically significant.

### Interim analyses {21b}

This is described in section Composition of the data monitoring committee, its role and reporting structure {21a}.

### Methods for additional analyses (e.g., subgroup analyses) {20b}

Subgroup analyses will not be conducted in this study. However, the primary and selected secondary (e.g., mortality) outcomes will be reported by sex.

### Methods in analysis to handle protocol non-adherence and any statistical methods to handle missing data {20c}

All data will be thoroughly checked for completeness and all efforts will be made to recover/locate missing it first before multiple imputation is used.

### Plans to give access to the full protocol, participant level-data and statistical code {31c}

The full study protocol will be made available via the trial website (https://www.research4babies.org). The study protocol has been also uploaded as a supplemental appendix to this article. At the end of the trial, the complete dataset and statistical codes will be available upon request.

## Oversight and monitoring

### Composition of the data monitoring committee, its role and reporting structure {21a}

A Data and Safety Monitoring Board (DSMB) will monitor the study to: (1) protect all study patients, (2) safeguard the interests of all study patients, (3) monitor the overall conduct of the trial, (4) advise the investigators to protect the integrity of the trial, and (5) supervise the conduct and analysis of all interim analyses. To its end the DSMB will receive regular reports from the trial on any injuries or adverse events, any developments that jeopardize the continued success of the trial, and data by which to accomplish the evaluation of pre-determined early stopping rules. Serious Adverse Events to be reported (mortality) will be sent within 72 hours to the DSMB; reports of other/less serious adverse events and recruitment will be sent monthly; demographics and adverse events (including pneumothorax, periventricular leukomalacia and intraventricular hemorrhage grade 3 or higher according to Papile^24^ will be included with the interim and final safety and efficacy analyses. The DSMB will perform interim safety analysis every 3 months to review the primary outcome of ROSC and serious adverse events. At the discretion of the DSMB further interim analyses can be requested. Members of the DSMB are Professor Gary Weiner (current Co-Chair of the Neonatal Task Force within the International Liaison Committee on Resuscitation, Professor Vishal Kapadia (Neonatal Resuscitation Program Co-Chair), and Professor Karel Allegaert (Expert in Neonatal Pharmacology).

### Adverse event reporting and harms {22}

The trial coordinator at the Royal Alexandra Hospital will maintain detailed records of all reported adverse events. Safety reporting will follow standards as per Health Canada regulations including adverse event, serious adverse event, and suspected unexpected serious adverse reaction. As infants who require CPR in the delivery room are a very seriously ill patient group. Most adverse events may be of a serious nature with or without the trial intervention, and both intervention groups are expected to have a very high proportion of serious adverse events. Serious adverse events to be recorded are therefore mortality within the delivery room (e.g., did not achieve ROSC or did achieve ROSC but care was withdrawn), and within the NICU (any mortality).

Adverse events we expect to be related to the application of the treatment guideline include: No ROSC leading to death, accidental displacement of the endotracheal tube or extubation, accidental displacement of venous or arterial catheters, use of Nitric Oxide for pulmonary hypertension, sepsis, pneumothorax, periventricular leukomalacia and intraventricular hemorrhage (grades 1–4) for preterm infants and cerebral infarction and hemorrhage for near-term and term infants.^24^

### Frequency and plans for auditing trial conduct {23}

The DSMB will review every 3 months for safety. By comparing cumulative data from study patients with comparative data from our SURV1VE-trials,^25^, ^26^ which included newborn infants who received CPR in the delivery room.

Stopping rules include:

1) An increased mortality in the Vasopressin group by 25% compared to the Epinephrine group at the predefined interim analysis every 6 months.
2) Increase in rate of morbidities including pneumothorax, intraventricular hemorrhage or the combination, in the Vasopressin group by 25% compared to the Epinephrine group at predefined interim analysis every 6 months.
3) Bayesian posterior probability of Vasopressin group being better than the control is less than 0.5 or greater than 0.98. (Posterior probability of Vasopressin arm to reduce time to ROSC by 10% or more compared to the control arm will be calculated. If this probability is less than 0.5, the trial will be stopped for futility. If the posterior probability is greater than 0.98, DSMB will consider the trial to be stopped for superiority.

### Plans for communicating important protocol amendments to relevant parties (e.g., trial participants, ethical committees) {25}

The sponsor/principal investigator and the ethics committees can make decisions about trial discontinuation. If the trial is terminated or suspended the parents of all trial participants will be informed and appropriate follow-up will be assured. If sponsor/principal investigator terminates or suspends the trial the relevant ethics committees will be provided with a detailed written explanation of the termination or suspension.

The sponsor/principal investigator can, upon completion of the analysis of the reason(s) for a suspension, decide to lift the suspension when the necessary corrective actions have been implemented. The investigators and ethics committees will be notified and provided with the relevant data supporting the decision.

### Dissemination plans {31a}

The trial was registered on ClinicalTrials.gov (NCT05738148) prior randomization of the starting intervention. Attempts will be sought to publish protocol, all results, positive, neutral, as well as negative, in peer-reviewed international journals. Authorship will be determined according to the International Committee of Medical Journal Editors. Attempts will be made to publish a list of all investigators with their contributions in all publications.

## Ethics and dissemination

### Ethics approval and consent to participate {24}

The study protocol (Version 1.2, May 4, 2023) was approved by the University of Alberta Research Ethics Office on June 15, 2023. We will obtain individual consent after birth for data inclusion in the trial as per Tri-Council Policy Statement in Human Research guidelines for research in “Individual Medical Emergencies”.^19^ For these infants to be included in the trial, consent will be sought from the parents as soon as possible after birth to use the data obtained.^18^ If an infant does not survive the delivery room, every effort will be made during the bereavement to i) explain and discuss the study with parents, ii) parents have ample time to ask questions, and we will ask their permission to retain the data collected to be included in the trial. If parents wish not to participate, all data will be deleted.

### Consent for publication {32}

Not applicable.

### Availability of data and materials {29}

The trial dataset will be stored and maintained at the Royal Alexandra Hospital, where it will be accessible to all trial investigators for quality monitoring. Other access to the study data will be available by request to the principal investigator. Data requestors seeking to use trial data to generate new publications or presentations will be asked to submit a proposal that will be reviewed by the principal investigator and co-investigators.

## Funding

This work was supported by a grant from the Neonatal Resuscitation Program of the Canadian Pediatric Society.

## Strengths and limitations of this study

• This trial will use an open label, cluster, single centre randomized controlled trial (RCT) with two alternative cardiovascular supportive medications in the delivery room.
• This trial will examine the differences in time to return of spontaneous circulation and clinical outcome measures in asphyxiated newborns treated with epinephrine vs. vasopressin.
• This trial will be the first neonatal clinical trial to examine the efficacy of vasopressin in the infants’ receiving CPR in the delivery room.
• Due to the low incidence of newborns requiring cardiopulmonary resuscitation at birth, the expected number of participants is expected to be approximately 20.

## CRediT authorship contribution statement

**M. Ramsie:** Conceptualization, Methodology, Writing – original draft, Writing – review & editing. **P.-Y. Cheung:** Conceptualization, Funding acquisition, Methodology, Writing – review & editing. **B. Law:** Conceptualization, Funding acquisition, Methodology, Writing – review & editing. **G.M. Schmölzer:** Conceptualization, Funding acquisition, Methodology, Writing – review & editing.

## Declaration of Competing Interest

The authors declare that they have no known competing financial interests or personal relationships that could have appeared to influence the work reported in this paper.
