# Supplementary material for: Vasopressin versus epinephrine during cardiopulmonary resuscitation of asphyxiated newborns: A study protocol for a prospective, cluster, open label, single-center, randomized controlled phase 2 trial – The VERSE-Trial
Source: Resusc Plus. 2023 Aug 31;16:100459. doi: 10.1016/j.resplu.2023.100459 (PMC10474318; doi:10.1016/j.resplu.2023.100459)
Supplement: Supplementary data 1 [file mmc1.docx]

**
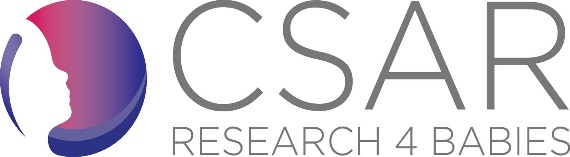
**

**Protocol**

**Vasopressor-Trial**

Epinephrine vs. Vasopressin during cardiopulmonary resuscitation of asphyxiated n**e**wborns – a cluster randomized controlled phase 2 trial

| **Protocol:** | Protocol Vasopressor |
| --- | --- |
| **Trial phase** | Phase 2 |
| **Protocol version:** | **Version 1.3** |
| **Protocol date:** | **August 4^th^, 2023** |
| **Study site:** | Royal Alexandra Hospital |
| **Sponsor:** | Governors of the University of Alberta |
|  |  |
| **Name and title of the person:** | Georg Schmölzer |
|  |  |

# Protocol Summary

| **Study Title** | Epinephrine vs. Vasopressin during cardiopulmonary resuscitation of asphyxiated n**e**wborns – a cluster randomized controlled phase 2 trial |
| --- | --- |
| **Population** | Newborns requiring chest compression and cardiovascular supportive medication in the delivery room. |
| **Primary Objective** | Vasopressin will improve short- and long-term outcomes in preterm and term newborns |
| **Primary Hypothesis** | When using Vasopressin during cardiopulmonary resuscitation (CPR), the time needed to achieve return of spontaneous circulation (ROSC) compared to epinephrine will be reduced in asphyxiated newborns. |
| **Design and Sample Size** | Prospective single-center randomized controlled trial (RCT) with two alternative cardiovascular supportive medications. 10-20 infants |
| **Inclusion Criteria** | Newborns requiring chest compression and cardiovascular supportive medication in the delivery room. |
| **Exclusion Criteria** | a) Congenital abnormality  b) Condition that might have an adverse effect on breathing or ventilation (e.g. congenital diaphragmatic hernia)  c) Congenital heart disease requiring intervention in the neonatal period)  d) Parents’ refusal to give consent to this study |
| **Efficacy Endpoints** | **Primary:**   - Time to achieve return of spontaneous circulation defined as a heart rate of >60/min for 60sec   **Secondary:**   - All mortality prior to discharge from hospital - Delivery room interventions - Admission temperature - Use of therapeutic cooling - Mechanical ventilation - Use of inotropes - Infection/sepsis - Necrotizing enterocolitis - Pneumothorax - Bronchoplumonary dysplasia - Retinopathy of prematurity - Brain injury as indicated by abnormal neuroimaging - Seizure |
| **Safety Evaluations** | Adverse events |
| **Statistical Methodology** | The final analysis will be conducted after the study is completed, unblinded, and the database is released for analysis. All analyses will be performed using IBM SPSS Statistics Ver. 24 (IBM Corp.) and SAS version 9.4 (SAS Institute Inc.) or later. |
|  |  |
| **Clinical Center** | Royal Alexandra Hospital |
| **Enrollment Period** | 2 years |
| **Study Duration** | 3 years |
| **Webpage** | http://www.research4babies.org |
| **ClinicalTrials.Gov Trial** | NCT05738148 |

List of abbreviations

CC - Chest compression

DR - Delivery room

CPR - Cardiopulmonary resuscitation

ROSC - Return of spontaneous circulation

C:V ratio - Compression to ventilation ratio

DBP - Diastolic blood pressure

CCaV - Continuous chest compression with asynchronous ventilations

V_T_ - Tidal volume

RN - Registered Neonatal Nurse

RRT - Registered Respiratory Therapist

NNP - Neonatal Nurse Practitioner

PIP - Peak inflation pressure

PEEP - Peep expiratory pressure

ECO_2_ - Exhaled carbon dioxide

eCRF - electronic Case Report Forms

SD - Standard deviation

IQR - Interquartile range

SAE - Serious adverse event

DSMB - Data and Safety Monitoring Board

GCP - Good Clinical Practice

NIRS -Near Infrared Spectroscopy

aEEG -amplitude integrated electroencephalography

# Trial design


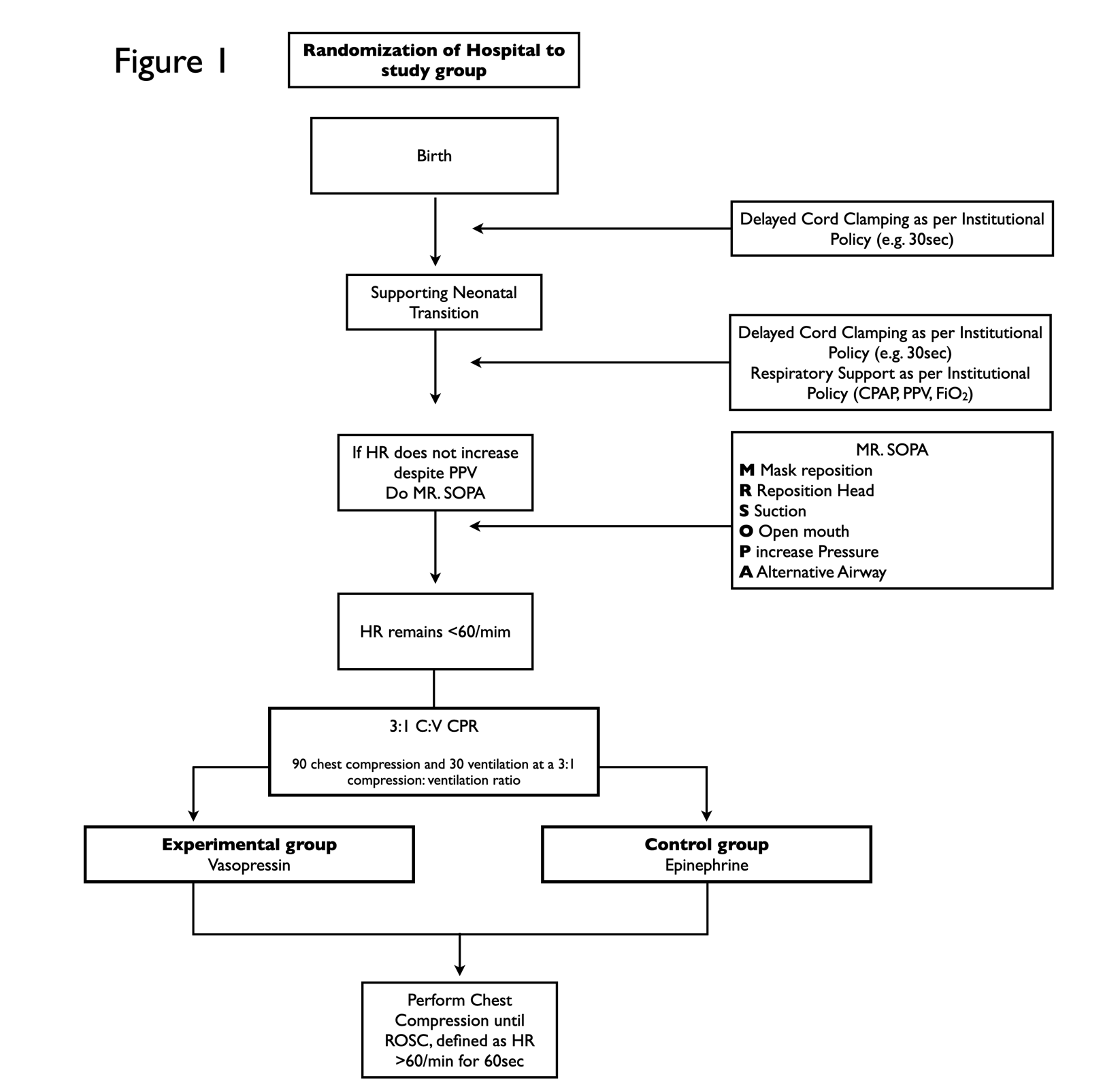


# Background

Most newborn infants successfully make the transition from fetal to neonatal life without help[1]. Between 10-20% of newborns (13-26 million worldwide) need respiratory support[2,3]. In the delivery room, 0.1% of term infants and 10-15% of preterm infants (2-3 million worldwide) need **cardiopulmonary resuscitation (CPR)**[2,3], defined as chest compressions (CC), 100% oxygen, and administration of the vasopressor drug, epinephrine. Despite receiving CPR, **approximately 1 million of these newborns die every year worldwide**. Newborn infants receiving extensive CPR in the delivery room have a high incidence of mortality (41%) and short-term neurologic morbidity (e.g., 57% hypoxic-ischemic encephalopathy and seizures)[4–7]. Further, newborns receiving epinephrine receiving epinephrine but with no signs of life at 10 minutes after birth had an 83% mortality rate and 93% of survivors will have moderate-to-severe disability[5]. **The poor prognosis associated with receiving epinephrine in the delivery room raises questions about whether using a specifically tailored vasopressor during neonatal CPR could improve outcomes.**

The infrequent need for CC and epinephrine during neonatal resuscitation, coupled with an inability to consistently anticipate which newborn infants are at high risk of requiring extensive CPR, explains the ongoing lack of high quality evidence (i.e., large randomized clinical trials) to better guide healthcare providers in their resuscitative effort. Guidelines for neonatal resuscitation recognized the lack of neonatal data and extrapolate data from studies with adults and adult/pediatric animal studies. Those data may not apply wholly to the neonatal population^2^. Therefore, we need neonatal data to determine the optimal vasopressor therapy during neonatal resuscitation.

***Primary objective:***  To compare vasopressin and epinephrine during neonatal CPR.

# Hypotheses to be tested

***Primary hypothesis:*** In newborns who require CPR does Vasopressin compared to Epinephrine reduce time to achieve return of spontaneous circulation defined as a heart rate of >60/min for 60sec .

# Knowledge to date

Incidence of asphyxia in preterm and term newborns varies between 1-9/1000 and 1–2/1000 live births and represents the third most common cause of neonatal death[8,9]. Asphyxia results from failure of placental gas exchange before delivery (e.g., abruption, chorioamnionitis) or deficient pulmonary gas exchange immediately after birth (e.g., apnea, respiratory distress syndrome). Asphyxia is impaired gas exchange with simultaneous hypoxia and hypercapnia. In >80% of asphyxiated neonates, it leads to mixed metabolic and respiratory acidosis plus dysfunction of one or more organ systems (including depressed myocardial function leading to cardiogenic shock, pulmonary hypertension, and ultimately cardiac arrest) [8,9]. The underlying etiology of bradycardia, and ultimately cardiac arrest, in neonates, results from severe hypoxemia, metabolic acidosis, and vascular compromise. This contrasts with the etiology most in adults, where onset of arrhythmias is followed by abrupt cessation of cardiac output in the setting of well-oxygenated blood. Ventilation is therefore more likely to be beneficial in neonatal CPR than in adult CPR[2,3].

In the asphyxiated and severely acidotic newborn, the vascular bed is maximally vasodilated with very low systemic vascular resistance. Providing CC will serve to mechanically pump the blood through the body until the myocardium becomes sufficiently oxygenated to maintain adequate output[10–17]. Optimized CC can generate ~30% of normal organ perfusion, with preferential (>50%) perfusion to the heart and brain[18]. The administration of systemic vasoconstrictors (epinephrine) induces intense peripheral vasoconstriction resulting in elevated systemic vascular resistance, enhanced diastolic blood flow, and increased coronary perfusion pressure (CPP) to improve coronary blood flow[15,16,19–22]. However, in severely acidotic hemodynamically compromised lambs’ intravenous epinephrine administration at 0.01 mg/kg did not improve cardiac output, heart rate or blood pressures.

Current neonatal resuscitation guidelines recommend administration of epinephrine once CPR has started at a dose of 0.02mg/kg preferably given intravenously (i.v.), with repeated doses every 3 min until ROSC[2,3]. These recommendations are based on adult animal data, because neonatal data are lacking.

**Epinephrine**, is an endogenous catecholamine with high affinity for α_1_, α_2_, β_1_, and β_2_-receptors present in cardiac and vascular smooth muscle[23,24]. Epinephrine causes vasoconstriction via stimulation of α_1_-receptors present in vascular smooth muscle, stimulation of α_2_-receptors causes presynaptic inhibition of norepinephrine release in the central nervous system and coronary vasoconstriction. Through β_1_-receptors, epinephrine increases heart rate (chronotropy), conduction velocity (dromotropy), contractility (inotropy), and rate of myocardial relaxation (lusitropy). β_2_-receptor stimulation leads to smooth muscle relaxation and in the myocardium increases contractility[24–29]. However, epinephrine also increases myocardial oxygen demand and respiratory and metabolic acidosis, a common occurrence during neonatal asphyxia, and inhibits hemodynamic responses (e.g., aggravated hypertension, or tachycardia after ROSC)[30]. Furthermore *in vivo* effects of epinephrine depend on the i) dose of epinephrine, ii) number of receptors available on target tissues, iii) affinity of these receptors, and iv) local target tissue environments. Neonatal animal studies reported that 85% of asphyxiated piglets with cardiac arrest will require vasopressors to achieve ROSC[24,31–34]. There are some methodological flaws in these studies including i) these studies examined CC and reported vasopressor use as secondary outcomes only, ii) used a sheep model, which is not an ideal model[35,36], and iii) used the same dose in all studies. In addition, Sobotka *et al*[32] reported that epinephrine administration (0.01 mg/kg) was a prerequisite for achieving ROSC, which occurred between 7-124sec after epinephrine administration in a transitional near-term lamb model of asphyxia-induced bradycardia. CC alone does not generate a sufficient diastolic blood pressure (a proxy for coronary artery perfusion pressure) to achieve ROSC[18]. Similarly, dp/dt (an assessment of diastolic function during isovolumic relaxation) only increases after epinephrine administration (0.02 mg/kg) compared to CC alone, which is associated with an increase in diastolic function, hence improved coronary artery perfusion pressure, which is a pre-requirement for ROSC[31]. Similarly, Halling *et al* reported that 24/30 newborn infants required an average of 3 doses of epinephrine to achieve ROSC[37].

Alternatively, **vasopressin***,* an antidiuretic hormone with vasoactive action through V1 receptor activation, is beneficial due to its postulated effects including combined pulmonary vasodilation and systemic vasoconstriction, not affected by respiratory and metabolic acidosis, and no increase in myocardial oxygen demand[23,38]. Currently, a single dose of vasopressin at 40 international units (IU) is recommend during adult CPR[39], which is supported by several randomized trials[40]. Evidence from large randomized trials in adults reported that vasopressin is superior to epinephrine when cardiac arrest was caused by primary asystole[40,41]. Wenzel *et al* compared vasopressin and epinephrine during out-of-hospital cardiac arrest in adults and reported similar rates of hospital admission in patients with ventricular fibrillation (46% vs. 43%) or pulseless electrical activity (34% vs. 31%)[41]. However, among patients with asystole, vasopressin was associated with significantly higher rates of hospital admission (29% vs. 20% p=0.02) and hospital discharge (5% vs. 2%, p=0.04). ***This suggests that vasopressin might be beneficial when asystole is the leading cause for cardiac arrest, when compared to that due to ventricular fibrillation or PEA.***

Vasopressin may therefore be beneficial during neonatal CPR because in newborn infants i) asphyxia results primarily in non-shockable rhythm (asphyxia (40-45%) or pulseless electrical activity (40-50%), rather than ventricular fibrillation <5%)[2,3,42–44], Further, pulmonary vascular resistance is characteristically more prominent in newborns. Vasopressin’s combined properties as pulmonary vasodilator and systemic vasoconstrictor properties of vasopressin might make it an ideal support drug in this context. However, evidence is limited on vasopressin effectiveness in pediatric or neonatal patients[45,46]. Duncan *et al* reported that only 5% of the 1293 pediatric patients received vasopressin during in-hospital cardiac arrest[45]. Although. patients who received vasopressin had a significantly longer duration of cardiac arrest (median 37 vs. 24min, p=0.004) and a longer time to ROSC, their survival at 24 hours or at discharge was similar to patients receiving epinephrine[45]. A recent feasibility study compared vasopressin (0.8IU/kg) after an initial epinephrine dose in patients <18 years of age (n=10) to ≥ two doses of epinephrine[46]. Patients who received vasopressin had increased 24-hr survival (80% vs. 30%, odds ratio (OR) (95%CI) 9.3 (1.5-57.7)), with similar time to ROSC, survival to hospital discharge, and neurologic status at discharge. Until now only one study has compared vasopressin with epinephrine in a neonatal piglet model of cardiac arrest[47]. The study reported higher survival rates with vasopressin vs. epinephrine [16/20 vs. 11/24 (p<0.05)] with less myocardial necrosis on autopsy.

Both asphyxiated piglets and newborn infants require epinephrine to achieve ROSC[4],21,23,24,29,51,52]. Further, there is only a 20% success rate after a single dose of i.v. epinephrine, with multiple doses needed by many newborns[32]. During asphyxiated in pediatric patients (~6 years of age) there was no improvement in ROSC but increased mortality after epinephrine administration[45,46]. Thus, an alternative vasopressors therapy might improve outcomes. In adult patients, vasopressin compared to epinephrine was associated with increased rates of ROSC [OR (95% CI) 1.70 (1.17, 2.47), p=0.005] and higher long-term survival [OR (95% CI) 1.80 (1.04, 3.12), p=0.04][103]. Therefore, vasopressin might reduce time to ROSC and improve outcomes for asphyxiated newborn infants. While there are several animal and neonatal cohort studies examining epinephrine [21,23,24,29,51,52,26,27,100-102], studies examined vasopressin are lacking.

| Table 1 | Mean ROSC time (sec) | # Survival | Survival time (min) |
| --- | --- | --- | --- |
| Vaso 0.2 (n=8) | 115(35) | 7/8 | 240 |
| Vaso 0.4 (n=8) | 149(86) | 7/8 | 240 |
| Vaso 0.8 (n=8) | 121(44) | 6/8 | 162 |
| Epi 0.02 (n=8) | 342(194) | 6/8 | 210 |

We compared vasopressin and epinephrine in our established piglet model of neonatal asphyxia and observed that mean (SD) time to ROSC with epinephrine (0.02 mg/kg with 342 (194)sec, respectively, compared vasopressin (0.2, 0.4, and 0.8 IU/kg with 115(35) 149(86), and 121(44)sec), respectively.


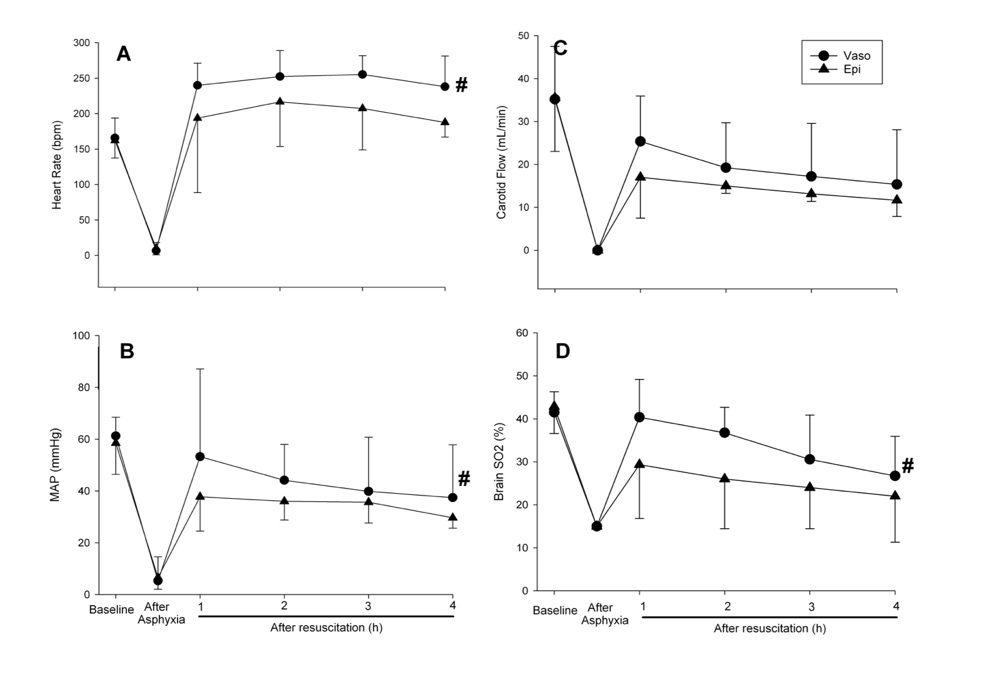
Furthermore, cardiovascular parameters including heart rate, carotid blood flow, mean arterial pressure (MAP), cerebral oxygenation (Brain SO_2_) we were also improved with Vasopressin, most effectively with 0.4, IU/kg.

Alternative administration of vasopressin endotracheal compared to epinephrine resulted in increases of up to 180% in diastolic blood pressure and up to 140% in systolic blood pressure, which are both essential for successful resuscitation.

These data suggest, that clinical data are needed to assess if vasopressin might be an alternative fir epinephrine.


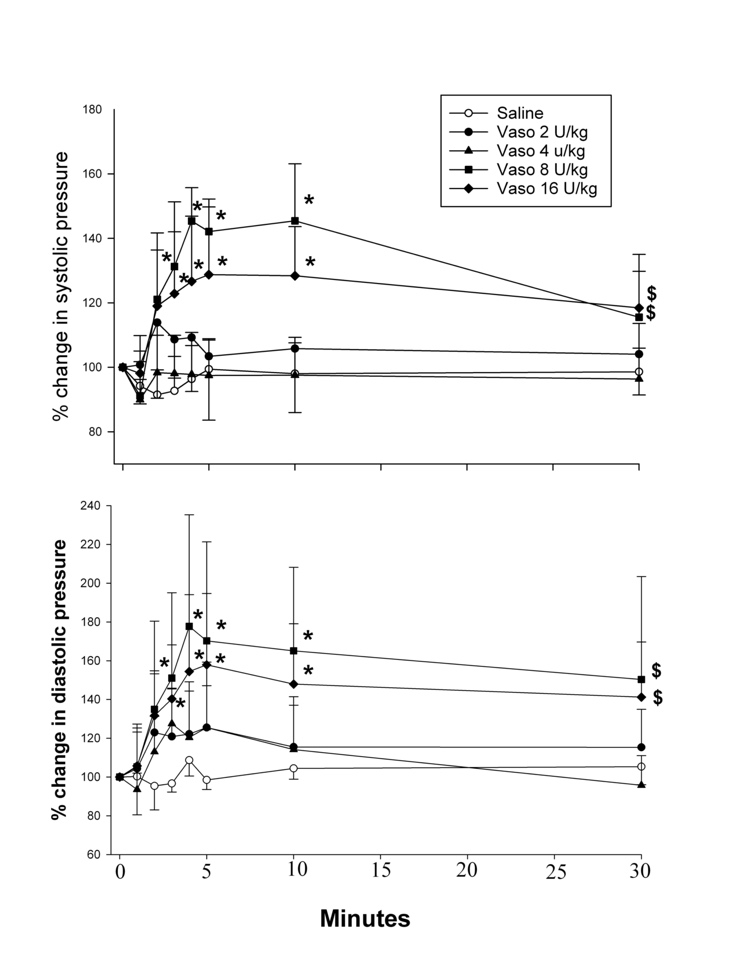


# Methodology

## Ethics

This cluster randomized clinical trial will be reviewed by the appropriate Research Ethics Office at the University of Alberta. We will request a deferred consent (see parental consent paragraph for details). Written consent will be sought from the parents of surviving infants as soon as possible after the birth so that acquired data could be utilized for research. For those infants who do not survive, we will request a waiver of consent.

## Inclusion criteria

All infants (term or preterm infants) requiring CC + vasopressor in the delivery room will be eligible for the trial.

## Exclusion criteria

Infants will be excluded if they have a congenital abnormality or condition that might have an adverse effect on breathing or ventilation (e.g., congenital diaphragmatic hernia), or congenital heart disease requiring intervention in the neonatal period. Infants will be also excluded if their parents refuse to give consent to this study.

## Patient discontinuation and withdrawal

The participant’s parents are free to withdraw the participant from the intervention or from the Vasopressor trial entirely until the baby has been discharged from the hospital, and this will not have any consequences for the participant’s further treatment. When possible, the parents will be asked if they will allow their infant to participate in the remaining follow-up assessments, and allow their infants already collected data to be used in a database, a registry, and/or a publication.

The attending clinician can withdraw the participant from the trial during neonatal cardiopulmonary resuscitation and revert to the standard treatment arm (epinephrine) as this is the only time the study drug will be administered. The reasons shall be documented. There are no pre-specified criteria for discontinuation of participants from the trial. The discontinuation of participants in the trial will not result in replacement with new participants.

## Randomization

At the beginning of the trial, the Royal Alexandra site will be randomized to either start with Vasopressin group (“intervention group”) or Epinephrine group (“control group”) for the first year. For the second year, the intervention group will be changed to control or vice versa.

## Sample Size and Power calculation

This will be the first trial ever to compare two medications during neonatal chest compression in the delivery room. Therefore, we can provide an adequate sample size calculation, and will aim to include as all infants requiring epinephrine or vasopressin in the delivery room with the 2-year time frame of the trial. In previous years, we have had between 5-10 infants per year requiring chest compressing and vasopressor, which we expect will be similar during the trial.

## Blinding

There will be no blinding during the recruitment phase as each year of the trial is allocated to one treatment arm and therefore the drug is known to the clinical team. The outcome assessor will be unaware of the group allocation. This blinding will be maintained until the data is locked for the final analysis and un-blinded.

## Technique of Resuscitation

Infants will only be eligible if they require CC + vasopressor (epinephrine or vasopressin). The methods described below also describe resuscitation interventions for infants prior to the start of chest compressions.

## Composition of the Resuscitation Team

The members of the resuscitation team will be site specific (for example in Edmonton it consists of a Registered Neonatal Nurse (RN), Respiratory Therapist (RT), Pediatric Resident or Neonatal Sub-Specialty Resident (Fellow) or Neonatal Nurse Practitioner (NNP) and/or Neonatologist). The most senior team member will act as the team leader, who will manage the protocol and guide the intervention during the trial.

## Description of general interventions

*Term infants*

The initial steps of the resuscitation will be according to the current neonatal resuscitation guidelines[2,3]. Cord management should be performed as per local hospital policy (standard hospital practice guideline) at participating sites where eligible. Afterwards, dry and stimulate the infant, and open the airway. If heart rate is <100/min, the infant is gasping or has apnea, start positive pressure ventilation. At the same time consider attaching a pulse oximeter and consider applying electrocardiography. If heart rate remains <100/min, perform MR. SOPA (**M**ask is tightly applied to the face, **R**e-position the head into the “sniffing” orientation, **S**uction the nares and the pharynx, **O**pen the mouth, **P**ressure of PPV can be increased to a max of 40 cm H_2_O, **A**lternate airway (endotracheal tube or Laryngeal Mask Airway). If heart rate is <60/min or remains <60 despite **all steps** of MR. SOPA including alternate airway, start chest compressions and increase to 100% oxygen.

*Preterm infants*

The initial steps of the resuscitation will be according to the current neonatal resuscitation guidelines[2,3] . Cord management should be performed as per local hospital policy (standard hospital practice guideline) at participating sites where eligible. Immediately afterwards, preterm infants (according to local hospital policy - standard hospital practice guideline) will be placed (without drying) in a polyethylene bag under radiant heat, whereas infants >28^+0^ weeks will be dried and placed under radiant heat.

A pulse oximeter will be used to monitor oxygen saturation immediately after birth and oxygen delivery will be guided by published norm values for oxygen saturation in preterm infants[48].

If heart rate is <100/min, the infant is gasping or has apnea, start positive pressure ventilation. Also consider applying electrocardiography at that time. If heart rate remains <100/min, perform MR. SOPA (**M**ask is tightly applied to the face, **R**e-position the head into the “sniffing” orientation, **S**uction the nares and the pharynx, **O**pen the mouth, **P**ressure of PPV can be increased to a max of 30 cm H_2_O, **A**lternate airway (endotracheal tube or Laryngeal Mask Airway). If heart rate is <60/min or remains <60 despite all steps of MR. SOPA start chest compression and increase to 100% oxygen.

## Mask Ventilation

The clinical team will determine if an infant requires mask ventilation. If mask ventilation is required, it should be provided with a T-Piece device (e.g. Fisher & Paykel, Auckland, New Zealand or Giraffe Warmer, GE Health Care, Burnaby, BC, Canada), and appropriate face mask with default settings for peak inflation pressure (PIP) of 24 cmH_2_O, a peep expiratory pressure (PEEP) of 6 cm H_2_O, and a gas flow rate of 10L/min using a ventilation rate of 40-60 inflations/min.

## Cardiopulmonary Resuscitation

If heart rate is <60/min or remains <60/min despite all steps of MR. SOPA chest compressions is started. Prior to the start of CC intubation should be considered. However, in cases of intubation failure it is possible to start CC during mask ventilation. Compressions will be performed using the 2-thumb encircling technique as recommended by current resuscitation guidelines[2,3].

## Interventions

*“Epinephrine group”*

Epinephrine will be administered according to current resuscitation guidelines either via umbilical vein catheter (0.02 mg/kg per dose) or via endotracheal tube (0.1 mg/kg) every three to five minutes as needed[2,3]. Chest compressions and epinephrine will be continued until ROSC.

*“Vasopressin group”*

Vasopressin will be via umbilical vein catheter (0.4 IU/kg per dose – first line) or alternatively via an endotracheal tube (8 IU/kg) every three to five minutes as needed with a maximum of two doses if there is no ROSC [2,3] After that, the clinical team must convert to give epinephrine (0.02 mg/kg per dose) as long as CPR is ongoing (opt-out rule).

**Treatment period**

Epinephrine is standard of care during neonatal CPR and therefore there is no treatment period. Epinephrine is given every 3-5 minutes during active CPR.

Vasopressin (intervention group), is only given during ongoing CPR and can only be given two times 3-5 minutes apart. The treatment period is no longer than 10 minutes.

## Determination of ROSC

ROSC will be defined as an increase in heart rate >60/min for 60sec determined by auscultation of the heart. Return of spontaneous circulation will be assessed after 60sec of a heart rate of >60/min.

## Discontinuing Resuscitation

Deciding how long resuscitative efforts should continue in any of the study infants will be solely at the discretion of the clinical team in accordance with the current neonatal resuscitation guidelines and per local hospital policy (standard hospital practice guideline).

**Medication permitted**

All medications are permitted before and during the trial. Epinephrine can be given as a rescue treatment during ongoing CPR and two doses of vasopressin have been given (see vasopressin group – opt-out-rule).

# Outcome measures

## Primary outcome

Time to ROSC will be defined as an increase in heart rate >60/min for 60sec determined by auscultation of the heart. Return of spontaneous circulation will be assessed after 60sec of a heart rate of >60/min.

## Secondary outcomes

Secondary outcomes amongst others will include neonatal mortality (Neonatal death <28 days) and morbidities, for example, rates of brain injury (reported either via magnetic resonance imaging (MRI) or head ultrasound), results of amplitude-integrated electroencephalography (aEEG) monitoring until normalization of background patterns and occurrence of sleep-wake cycling, DR interventions (including mask ventilation, intubate, chest compression), admission temperature, use of therapeutic cooling, mechanical ventilation, pneumothorax, use of inotropes, infection/sepsis, intraventricular hemorrhages, necrotizing enterocolitis, bronchopulmonary dysplasia, retinopathy of prematurity. Targeted Neonatal Echocardiography TnECHO to assess cardiac function in the first 24 hours.

Infants who received chest compression at birth receive routine follow-up at 18 and 24 months after birth at the Infant Follow-up Clinic at the Glenrose Rehabilitation Hospital. This follow-up exam is standard of care and assesses the presence of a major neurodevelopmental outcome defined as any one of: (i) cerebral palsy with an inability to walk unassisted; (ii) major developmental delay involving cognition or speech or (iii) visual (cannot fixate/ legally blind, or corrected acuity <6/60 in both eyes), or hearing impairment (requiring a hearing aid or cochlear implants). These outcomes have become a standard expectation of evaluation in neonatal trials.

# Outcome assessment tools

Mortality: All causes of mortality will be recorded. Cerebral injury will be assessed with either cerebral ultrasound or cerebral MRI if performed prior to discharge. If an infant die prior to any neuro-imaging, a request for autopsy should be made. Autopsy could include imaging alone or full autopsy. Morbidity: Case history until discharge or term age (for preterm infants). Secondary outcome parameters: Case history and output from feedback devices.

# Compliance with the Protocol

The clinical investigation will be conducted in compliance with this protocol. Modifications to the protocol will not be implemented without agreement from the Principal Investigators and relevant ethics committee approval are obtained.

Investigators are not allowed to deviate from the protocol except as specified above. Any major or safety related deviations will be recorded, analyzed and the ethics committees notified. If an investigator refuses to comply with the protocol, he/she will be disqualified.

# Data collection

Infants will be recruited over a period of 24 months. Approximately another six months will be required to collect hospital data on all infants enrolled. Resuscitation data will be collected on a standard form (Neonatal Resuscitation Record) that will form part of each infant’s hospital record. The Neonatal Resuscitation Record should be completed by the clinical team attending the resuscitation. Other medical data on each infant will be collected on an electronic Case Report Form (eCRF). The eCRF will be designed in collaboration with Women and Children's Health Research Institute, University of Alberta, Edmonton, Canada. Data will be entered into REDCap database from each site within one week after discharge or death of an infant. Long-term follow-up will be entered within two weeks after examination. All information entered into REDCap database will be used for analysis.

# Data analysis

The Clinical Research Informatics Core and Biostatistics Core of the Women and Children’s Health Research Institute, at the University of Alberta, will do data analysis. Additional information about the storage, management and analysis of study data is available.

All analyses will be carried out according to the intention-to-treat principle. Statistical analyses will be performed using IBM SPSS Statistics Ver. 24 (IBM Corp.) and SAS version 9.4 (SAS Institute Inc.) or later.

**Interim Analysis**

There will be no interim safety analysis during this cluster trial as this is a single site trial.

**Final Analysis**

**Primary Analysis**

The primary outcome is the time to achieve ROSC defined as a heart rate of >60/min for 60sec. Data will be analyzed on an intention-to treat basis and will include all randomized participants. A per protocol analysis will also be conducted using the data from the actual allocation of participating infants. A survival analysis will be used to analyze the difference in time to ROSC between intervention and control groups. To account for cluster randomization, Cox proportional hazards regression with time to ROSC as an outcome and allocation group as an independent variable will be created. The analysis will be 2-sided and *p*-value < 0.05 will be considered statistically significant.

**Secondary Analysis**

The data will be presented as mean (standard deviation, SD) for normally distributed continuous variables and median (interquartile range, IQR) when the distribution is skewed. The clinical characteristics and outcome parameters will be compared using Student’s *t*-test for parametric and Mann-Whitney *U*-test for nonparametric comparisons of continuous variables, and χ^2^ for categorical variables. All *p*-values will be 2-sided and *p* <0.05 will be considered statistically significant.

**Assessment and documentation of safety events in the** Vasopressor **-trial**

The trial coordinator at the Royal Alexandra Hospital will maintain detailed records of all reported adverse events. Safety reporting from the Vasopressor-Trial will follow standards as per Health Canada regulations.

- An ADVERSE EVENT (AE) is any untoward medical occurrence in a patient or clinical trial participant administered a pharmaceutical product and that does not necessarily have a causal relationship with this treatment
- A SERIOUS ADVERSE EVENT (SAE) is any adverse event/adverse reaction that results in death, is life threatening, requires hospitalization, results in prolongation of existing hospitalization, results in persistent or significant disability or incapacity or is a birth defect
- A SUSPECTED UNEXPECTED SERIOUS ADVERSE REACTION (SUSAR) is an adverse reaction that is related to the drug that is both serious and unexpected

**The description of each AE on the CRF will include:**

- A description of the AE
- The onset date, duration, date of resolution
- Severity (mild, moderate or severe – what is the impact on the participant’s daily life?)*
- Seriousness (i.e., is it an SAE?)
- Any action taken, (e.g., treatment, follow-up tests)
- The outcome (recovery, death, continuing, worsening)
- The likelihood of the relationship of the AE to the study treatment (Unrelated, Possible, Probable, Definite)

Changes in the severity of an AE will be reported. AEs characterized as intermittent will be documented for each episode. All AEs will be followed to adequate resolution, where possible.

**Reporting of SAEs and SUSARs**

The investigator is responsible for reporting all SAEs and SUSARs occurring during the study to the Clinical Trial Coordinating Centre within 48 hours of the investigator becoming aware of the event using an SAE form. Furthermore, all SUSARs will be reported within 7 days to Health Canada as per section C.05.014 of the Food and Drug Regulations. In addition, all deaths will be reviewed (blinded to group allocation) by the DSMB after they occur; the DSMB will assign the likelihood of the death being related to the study intervention and whether the death was cardiovascular-related. SAEs must be reported until final hospital discharge or death.

Infants who require CPR in the DR are a very seriously ill patient group. Most adverse events may be of a serious nature with or without the Vasopressor-trial intervention, and both intervention groups are expected to have a very high proportion of serious adverse events (SAEs). Serious adverse events to be recorded are therefore mortality within the DR (e.g., did not achieve ROSC or did achieve ROSC but care was withdrawn), and within the NICU (any mortality).

## Expected adverse events

Adverse events we expect to be related to the application of the treatment guideline include: No ROSC leading to death, accidental displacement of the endotracheal tube or extubation, accidental displacement of venous or arterial catheters, use of Nitric Oxide for pulmonary hypertension, sepsis, pneumothorax, and intraventricular hemorrhage (grades 1-4)[49].

## Data and Safety Monitoring Board

A Data and Safety Monitoring Board (DSMB) will monitor the study to: (1) protect all study patients, (2) safeguard the interests of all study patients, (3) monitor the overall conduct of the trial, (4) advise the investigators in order to protect the integrity of the trial, and (5) supervise the conduct and analysis of all interim analyses. To its end the DSMB will receive regular reports from the trial on any injuries or adverse events, any developments that jeopardize the continued success of the trial, and data by which to accomplish the evaluation of pre-determined early stopping rules. Serious Adverse Events to be reported (mortality) will be sent within 72 hours to the DSMB; reports of other/less serious adverse events and recruitment will be sent monthly; demographics and adverse events (including pneumothorax, and intraventricular hemorrhage grade 3 or higher according to Papile[49]) will be included with the interim and final safety and efficacy analyses. The DSMB will perform interim safety analysis every 6 months to review the primary outcome of ROSC and SAEs.

At the discretion of the DSMB further interim analyses can be requested.

Members of the DSMB are Professor Gary Weiner (current Co-Chair of the Neonatal Task Force within the International Liaison Committee on Resuscitation (ILCOR), Professor Vishal Kapadia (Member of the Neonatal Task Force within ILCOR), and Professor Karel Allegaert (World Expert in Neonatal Pharmacology).

## Suspension or premature termination of the clinical investigation

The sponsor/principal investigator and the ethics committees can make decisions about trial discontinuation. If the trial is terminated or suspended the parents of all trial participants will be informed and appropriate follow-up will be assured. If sponsor/principal investigator terminates or suspends the trial the relevant ethics committees will be provided with a detailed written explanation of the termination or suspension.

The sponsor/principal investigator can, upon completion of the analysis of the reason(s) for a suspension, decide to lift the suspension when the necessary corrective actions have been implemented. The investigators and ethics committees will be notified and provided with the relevant data supporting the decision.

## Stopping rules

The DSMB will review every 3 months for safety. By comparing cumulative data form study patients with comparative data from our SURV1VE-trial, which included newborn infants who received chest compression in the delivery room and was completed in 2022.

Stopping rules include:

1) An increased mortality in the Vasopressin group by 25% compared to the Epinephrine group at the predefined interim analysis every 6 months.

2) Increase in rate of morbidities including pneumothorax, intraventricular hemorrhage or the combination, in the Vasopressin group by 25% compared to the Epinephrine group at predefined interim analysis every 6 months.

3) Bayesian posterior probability of Vasopressin group being better than the control is less than 0.5 or greater than 0.98. (Posterior probability of Vasopressin arm to reduce time to ROSC by 10% or more compared to the control arm will be calculated. If this probability is less than 0.5, the trial will be stopped for futility. If the posterior probability is greater than 0.98, DSMB will consider the trial to be stopped for superiority.

Since no statistical tests will be performed at interim analyses, type I error (alpha) does not need adjustment).

## Opt-out Rule

In any cases were during CPR, two doses of vasopressin were given and no ROSC, the clinical team must convert to giving a rescue dose of epinephrine of via umbilical vein catheter (0.02 mg/kg per dose)[2,3].

# Ethical Considerations

The Vasopressor-trial will be conducted in compliance with the guidelines of the Declaration of Helsinki in its latest form, the International Conference on Harmonization of Good Clinical Practice Guidelines. In case of modifications in the study protocol that are not merely of a formal nature but contain changes pertinent to the study participants, a renewed vote of the relevant ethics committees will be obtained. If applicable, the patients (parents) will be informed in the patient information and consent form about changes in the terms and conditions of the trial. The Vasopressor-trial will only start the randomization of participants after approval from the relevant ethics committees have been obtained.

## Parental consent

We aim to obtain individual consent after birth for data inclusion in the trial and follow-up as per Tri-Council Policy Statement in Human Research guidelines for research in "Individual Medical Emergencies". For these infants, consent will be sought from the parents as soon as possible after birth to use the data obtained.

Guidelines for a obtaining individual consent after birth for data inclusion in the trial and follow-up as laid down by Tri-Council Policy Statement state that:

1. “A serious threat to the prospective participant requires immediate intervention.”
   Infants participating in this trial will have cardiac arrest (or severe bradycardia), which in most cases is unforeseen prior to delivery; hence these infants will all need CPR and using chest compression will be therapeutic. However, only epinephrine (Epinephrine group) is routinely used around the world. It would not be feasible for a neonatal CPR study to ask permission prior to delivery from every parent delivering within the participating site.
2. “either no standard efficacious care exists or the research offers a realistic possibility of direct benefit to the participant in comparison with standard care.”

The currently used approach of epinephrine is extrapolated from animal studies and human adult studies. This will be the first pilot trial (Schmölzer PI) to examine an alternative drug, which is called vasopressin. The available animal evidence suggest that the interventional approach has the potential to improve standards of care.

1. “either the risk is not greater than that involved in standard efficacious care, or it is clearly justified by the prospect for direct benefits to the participant.*”* Resuscitation is therapeutic and the currently available animal evidence suggest that the interventional approach has no higher risk of harm compared to the current standard of care (Epinephrine group).
2. “the prospective participant is unconscious or lacks capacity to understand the risks, methods and purposes of the research project.” A woman in labor cannot give a valid informed consent to a research study. The parents are not considered to be capable of receiving and understanding information about the trial immediately following the birth of a severely compromised child. Thus, the parents will be informed as soon as possible after stabilization of the infant about the study and asked to consent to the use of data that have been collected on their child.
3. “third party authorization cannot be secured in sufficient time, despite diligent and documented efforts to do so;” The parents will be informed as soon as possible after birth/resuscitation/ROSC/death about the study and asked to consent to the use of data that have been collected on their child.
4. “No relevant prior directive by the participant is known to exist.”

The proposed consent approach is routinely used within DR research. In fact, the PI of this application has ample experience using this approach during neonatal resuscitation studies[50–54]. This study fulfills the criteria for obtaining individual consent after birth for data inclusion in the trial and follow-up in Europe, Canada and the United States. It also complies with the ethical requirements laid down by the National Health and Medical Research Council Australia for the ethical conduct of trials in neonatal intensive care research.

## Consent for non-survivors

Every effort will be made discuss the study with parents of non-surviving infant during bereavement. We will meet during bereavement with the parents to explain and discuss the study. Parents will have ample time to ask questions about the study. We will ask their permission during bereavement to retain the data collected to be included in the trial. If parents wish not to participate, all data will be deleted.

# Data management

## Data handling and archiving

Source data will be registered in the participant’s medical records/CRF and into the eCRF. A common web-based eCRF will be devised to enable a central database (Women and Children's Health Research Institute, University of Alberta, Edmonton, Canada). Data entry into the central database and handling of medical records is the responsibility of the investigators. After the establishment of a ’clean file’, the database will be locked; The data will be locked after completion of patient recruitment and data entry. After long-term follow-up data entry this portion of the database will locked. Data will be stored for statistical analysis at the Biostatistics Unit, Women and Children's Health Research Institute, University of Alberta, Edmonton, Canada. The trial database will hereafter be kept according to the respective national laws. After the end of trial, the data will be archived for 15 years according to good clinical practice guidelines. At each trial-site the data flow will be monitored according to the GCP principles by a locally appointed external monitoring committee. After completion of statistical data analysis, data will be pseudo-anonymized and stored at the University of Alberta, Edmonton, Canada.

## Data protection

The investigator(s) permits trial-related monitoring, audits, and regulatory inspection(s) by providing direct access to the source data and other relevant documents. Trial data will be handled according to regulations of the data protection agency in the respective countries.

## Quality assurance

The trial will be carried out in accordance with the Declaration of Helsinki in its latest form and the International Conference on Harmonization Good Clinical Practice (ICH-GCP) guidelines.

# Monitoring

The chief investigator consents to data evaluation being performed by the person in charge of monitoring in accordance with the monitoring plan, to ensure satisfactory data collection and adherence to the study protocol. Furthermore, the chief investigator states that he/she is willing to cooperate with this person and shall provide this person with all required information whenever necessary. This includes access to all documents related to the trial, including study-relevant medical files of patients in original form. The tasks of the investigator include maintenance of these patients' medical files as comprehensively as possible; this includes information concerning medical history, accompanying diseases, inclusion in the trial, data about visits, results of investigations, dispensing of medication, and adverse events. The monitor will also be permitted to perform data evaluation and draw comparisons with the relevant medical files in accordance with the standard operating procedures and ICH-GCP guidelines at predetermined intervals, to ensure adherence to the study protocol and continuous registration of data. All original medical reports required as sources for the information given in the CRF or the database shall be inspected. The study participants will have given their consent to such inspection by signing the consent form. The person in charge of monitoring is obliged to treat all information as confidential and to preserve the basic claims of the study participants in respect of integrity and protection of their privacy.

# Publication plan

The trial will be registered on ClinicalTrials.gov (NCT05738148) prior to the randomization of the first participant. Attempts will be sought to publish protocol, all results, positive, neutral, as well as negative, in peer-reviewed international journals. Authorship will be determined according to the International Committee of Medical Journal Editors. Attempts will be made to publish a list of all investigators with their contributions in all publications.

# References

[1] Aziz K, Chadwick M, Baker M, Andrews W. Ante- and intra-partum factors that predict increased need for neonatal resuscitation. Resuscitation 2008;79:444–52. https://doi.org/10.1016/j.resuscitation.2008.08.004.

[2] Wyckoff MH, Wyllie JP, Aziz K, Almeida MF de, Fabres J, Fawke J, et al. Neonatal Life Support: 2020 International Consensus on Cardiopulmonary Resuscitation and Emergency Cardiovascular Care Science With Treatment Recommendations. Circulation 2020;142:329–37. https://doi.org/10.1161/cir.0000000000000895.

[3] Aziz K, Lee HC, Escobedo MB, Hoover AV, Kamath-Rayne BD, Kapadia VS, et al. Part 5: Neonatal Resuscitation: 2020 American Heart Association Guidelines for Cardiopulmonary Resuscitation and Emergency Cardiovascular Care. Circulation 2020;142:1–27. https://doi.org/10.1161/cir.0000000000000902.

[4] Barber CA, Wyckoff MH. Use and Efficacy of Endotracheal Versus Intravenous Epinephrine During Neonatal Cardiopulmonary Resuscitation in the Delivery Room. Pediatrics 2006;118:1028–34. https://doi.org/10.1542/peds.2006-0416.

[5] Harrington DJ, Redman CW, Redman CW, Moulden M, Greenwood CE. The long-term outcome in surviving infants with Apgar zero at 10 minutes: a systematic review of the literature and hospital-based cohort. American Journal of Obstetrics and Gynecology 2007;196:463.e1-5. https://doi.org/10.1016/j.ajog.2006.10.877.

[6] Shah PS, Shah P, Tai KFY, Tai KFY. Chest compression and/or epinephrine at birth for preterm infants <32 weeks gestational age: matched cohort study of neonatal outcomes. Journal of Perinatology 2009;29:693–7. https://doi.org/10.1038/jp.2009.70.

[7] Soraisham AS, Lodha AK, Singhal N, Aziz K, Yang J, Lee SK, et al. Neonatal outcomes following extensive cardiopulmonary resuscitation in the delivery room for infants born at less than 33 weeks gestational age. Resuscitation 2014;85:238–43. https://doi.org/10.1016/j.resuscitation.2013.10.016.

[8] Gopagondanahalli KR, Li J, Fahey MC, Hunt RW, Jenkin G, Miller SL, et al. Preterm Hypoxic–Ischemic Encephalopathy. Frontiers in Pediatrics 2016;4:1–10. https://doi.org/10.3389/fped.2016.00114.

[9] Joynt C, Cheung P-Y. Cardiovascular Supportive Therapies for Neonates With Asphyxia — A Literature Review of Pre-clinical and Clinical Studies. Frontiers in Pediatrics 2018;6:786–16. https://doi.org/10.3389/fped.2018.00363.

[10] Halperin HH, Tsitlik J, Guerci AD, Mellits ED, Levin HR, Shi AY, et al. Determinants of blood flow to vital organs during cardiopulmonary resuscitation in dogs. Circulation 1986;73:539–50. https://doi.org/10.1161/01.cir.73.3.539.

[11] Rudikoff M, Maughan WL, Effron M, Fresson J, Weisfeldt ML. Mechanisms of blood flow during cardiopulmonary resuscitation. Circulation 1980;61:345–52. https://doi.org/10.1161/01.cir.61.2.345.

[12] Chandra N, Weisfeldt ML, Tsitlik J, Vaghaiwalla F, Snyder LD, Hoffecker M, et al. Augmentation of carotid flow during cardiopulmonary resuscitation by ventilation at high airway pressure simultaneous with chest compression. Am J Cardiol 1981;48:1053–63. https://doi.org/10.1016/0002-9149(81)90320-9.

[13] Chandra N, Rudikoff M, Weisfeldt MyronL. SIMULTANEOUS CHEST COMPRESSION AND VENTILATION AT HIGH AIRWAY PRESSURE DURING CARDIOPULMONARY RESUSCITATION. Lancet 1980;315:175–8. https://doi.org/10.1016/s0140-6736(80)90662-5.

[14] Higano ST, Oh JK, Ewy GA, Seward JB. The mechanism of blood flow during closed chest cardiac massage in humans: transesophageal echocardiographic observations. Mayo Clinic Proceedings Mayo Clinic 1990;65:1432–40.

[15] Berg RA, Sanders AB, Kern KB, Hilwig RW, Heidenreich JW, Porter ME, et al. Adverse Hemodynamic Effects of Interrupting Chest Compressions for Rescue Breathing During Cardiopulmonary Resuscitation for Ventricular Fibrillation Cardiac Arrest. Circulation 2001;104:2465–70. https://doi.org/10.1161/hc4501.098926.

[16] Kern KB, Hilwig RW, Berg RA, Sanders AB, Ewy GA. Importance of continuous chest compressions during cardiopulmonary resuscitation: improved outcome during a simulated single lay-rescuer scenario. Circulation 2002;105:645–9. https://doi.org/10.1161/hc0502.102963.

[17] Koehler RC, Tsitlik J, Chandra N, Guerci AD, Rogers MC, Weisfeldt ML. Augmentation of cerebral perfusion by simultaneous chest compression and lung inflation with abdominal binding after cardiac arrest in dogs. Circulation 1983;67:266–75. https://doi.org/10.1161/01.cir.67.2.266.

[18] Wyckoff MH, Berg RA. Optimizing chest compressions during delivery-room resuscitation. Seminars in Fetal and Neonatal Medicine 2008;13:410–5. https://doi.org/10.1016/j.siny.2008.04.012.

[19] Berg RA, Hilwig RW, Kern KB, Ewy GA. “Bystander” Chest Compressions and Assisted Ventilation Independently Improve Outcome From Piglet Asphyxial Pulseless “Cardiac Arrest.” Circulation 2000;101:1743–8. https://doi.org/10.1161/01.cir.101.14.1743.

[20] Berg RA, Ewy GA, Zuercher M, Hilwig RW, Sanders AB, Otto CW, et al. Improved Neurological Outcome With Continuous Chest Compressions Compared With 30:2 Compressions-to-Ventilations Cardiopulmonary Resuscitation in a Realistic Swine Model of Out-of-Hospital Cardiac Arrest. Circulation 2007;116:2525–30. https://doi.org/10.1161/circulationaha.107.711820.

[21] Wyckoff MH, Kapadia VS. Drugs during delivery room resuscitation - What, when and why? Seminars in Fetal and Neonatal Medicine 2013;18:357–61. https://doi.org/10.1016/j.siny.2013.08.001.

[22] Wyckoff MH, Perlman JM. Use of high-dose epinephrine and sodium bicarbonate during neonatal resuscitation: is there proven benefit? Clinics in Perinatology 2006;33:141-51-viii–ix. https://doi.org/10.1016/j.clp.2005.11.016.

[23] Kapadia VS, Wyckoff MH. Epinephrine Use during Newborn Resuscitation. Frontiers in Pediatrics 2017;5:97. https://doi.org/10.3389/fped.2017.00097.

[24] Pinto M, Solevåg A, OʼReilly M, Aziz K, Cheung P-Y, Schmölzer GM. Evidence on Adrenaline Use in Resuscitation and Its Relevance to Newborn Infants: A Non-Systematic Review. Neonatology 2017;111:37–44. https://doi.org/10.1159/000447960.

[25] PEARSON JW, REDDING JS. THE ROLE OF EPINEPHRINE IN CARDIAC RESUSCITATION. Anesthesia & Analgesia 1963;42:599–606.

[26] REDDING JS, PEARSON JW. Evaluation of drugs for cardiac resuscitation. Anesthesiology 1963;24:203–7. https://doi.org/10.1097/00000542-196303000-00008.

[27] Otto CW, Yakaitis RW, Blitt CD. Mechanism of action of epinephrine in resuscitation from asphyxial arrest. Critical Care Medicine 1981;9:321–4. https://doi.org/10.1097/00003246-198104000-00008.

[28] Kuznetsov V, Pak E, Robinson RB, Steinberg SF. β 2-Adrenergic Receptor Actions in Neonatal and Adult Rat Ventricular Myocytes. Circulation Research 1995;76:40–52. https://doi.org/10.1161/01.res.76.1.40.

[29] Devic E, Xiang Y, Gould D, Kobilka B. Beta-adrenergic receptor subtype-specific signaling in cardiac myocytes from beta(1) and beta(2) adrenoceptor knockout mice. Molecular Pharmacology 2001;60:577–83.

[30] PREZIOSI MP, ROIG JC, HARGROVE N, BURCHFIELD DJ. Metabolic acidemia with hypoxia attenuates the hemodynamic responses to epinephrine during resuscitation in lambs. Crit Care Med 2006;21:1901–7. https://doi.org/10.1097/00003246-199312000-00018.

[31] Wagner M, Cheung P-Y, Li ES, Lee T-F, Lu M, Olischar M, et al. Effects of epinephrine on hemodynamic changes during cardiopulmonary resuscitation in a neonatal piglet model. Pediatric Research 2018;83:897–903. https://doi.org/10.1038/pr.2017.316.

[32] Sobotka K, Polglase GR, Schmölzer GM, Davis PG, Klingenberg C, Hooper SB. Effects of chest compressions on cardiovascular and cerebral hemodynamics in asphyxiated near-term lambs. Pediatric Research 2015;78:395–400. https://doi.org/10.1038/pr.2015.117.

[33] Vali P, Sankaran D, Rawat M, Berkelhamer S, Lakshminrusimha S. Epinephrine in Neonatal Resuscitation. Children 2019;6:51–15. https://doi.org/10.3390/children6040051.

[34] Vali P, Chandrasekharan PK, Rawat M, Gugino SF, Koenigsknecht C, Helman J, et al. Evaluation of Timing and Route of Epinephrine in a Neonatal Model of Asphyxial Arrest. J Am Hear Assoc 2017;6:e004402-12. https://doi.org/10.1161/jaha.116.004402.

[35] Solevåg A, Cheung P-Y, Lie H, O’Reilly M, Aziz K, Nakstad B, et al. Chest compressions in newborn animal models: A review. Resuscitation 2015;96:151–5. https://doi.org/10.1016/j.resuscitation.2015.08.001.

[36] Hooper SB, Pas AB te, Polglase GR, Wyckoff MH. Animal models in neonatal resuscitation research: What can they teach us? Seminars in Fetal and Neonatal Medicine 2018;23:300–5. https://doi.org/10.1016/j.siny.2018.07.002.

[37] Halling C, Sparks JE, Christie L, Wyckoff MH. Efficacy of Intravenous and Endotracheal Epinephrine during Neonatal Cardiopulmonary Resuscitation in the Delivery Room. J Pediatr 2017;185:232–6. https://doi.org/10.1016/j.jpeds.2017.02.024.

[38] Antonucci R, Antonucci L, Locci C, Porcella A, Cuzzolin L. Current Challenges in Neonatal Resuscitation: What is the Role of Adrenaline? Pediatric Drugs 2018;33:1–12. https://doi.org/10.1007/s40272-018-0300-6.

[39] Neumar RW, Otto CW, Link MS, Kronick SL, Shuster M, Callaway CW, et al. Part 8: Adult Advanced Cardiovascular Life Support: 2010 American Heart Association Guidelines for Cardiopulmonary Resuscitation and Emergency Cardiovascular Care. Circulation 2010;122:S729–67. https://doi.org/10.1161/circulationaha.110.970988.

[40] Aung K, Htay T. Vasopressin for cardiac arrest: a systematic review and meta-analysis. 2005;165:17–24. https://doi.org/10.1001/archinte.165.1.17.

[41] Wenzel V, Krismer AC, Arntz HR, Sitter H, Stadlbauer KH, Lindner KH, et al. A comparison of vasopressin and epinephrine for out-of-hospital cardiopulmonary resuscitation. New England Journal of … 2004;350:105–13. https://doi.org/10.1056/nejmoa025431.

[42] Luong DH, Cheung P-Y, Barrington KJ, Davis PG, Unrau J, Dakshinamurti S, et al. Cardiac arrest with pulseless electrical activity rhythm in newborn infants: a case series. Archives of Disease in Childhood Fetal and Neonatal Edition 2019:fetalneonatal-2018-316087-3. https://doi.org/10.1136/archdischild-2018-316087.

[43] Luong DH, Cheung P-Y, Lee T-F, Schmölzer GM. Electrocardiography vs. Auscultation to Assess Heart Rate During Cardiac Arrest With Pulseless Electrical Activity in Newborn Infants 2018;6:S204-4. https://doi.org/10.3389/fped.2018.00366.

[44] Patel S, Cheung P-Y, Solevåg A, Barrington KJ, Kamlin COF, Davis PG, et al. Pulseless electrical activity: A misdiagnosed entity during asphyxia in newborn infants? Archives of Disease in Childhood-Fetal … 2018:fetalneonatal-2018-314907. https://doi.org/10.1136/archdischild-2018-314907.

[45] Duncan JM, Meaney P, Simpson P, Berg RA, Nadkarni V, Schexnayder S, et al. Vasopressin for in-hospital pediatric cardiac arrest: results from the American Heart Association National Registry of Cardiopulmonary Resuscitation. Pediatric Critical Care Medicine : A Journal of the Society of Critical Care Medicine and the World Federation of Pediatric Intensive and Critical Care Societies 2009;10:191–5. https://doi.org/10.1097/pcc.0b013e31819a36f2.

[46] Carroll TG, Dimas VV, Raymond TT. Vasopressin rescue for in-pediatric intensive care unit cardiopulmonary arrest refractory to initial epinephrine dosing. Pediatric Critical Care Medicine 2012;13:265–72. https://doi.org/10.1097/pcc.0b013e31822f1569.

[47] McNamara PJ, Engelberts D, Finelli M, Adeli K, Kavanagh BP. Vasopressin improves survival compared with epinephrine in a neonatal piglet model of asphyxial cardiac arrest. Pediatric Research 2014;75:738–48. https://doi.org/10.1038/pr.2014.38.

[48] Dawson JA, Kamlin COF, Vento M, Wong C, Cole TJ, Donath S, et al. Defining the reference range for oxygen saturation for infants after birth. Pediatrics 2010;125:e1340-7. https://doi.org/10.1542/peds.2009-1510.

[49] Papile L-A, Burstein J, Burstein J, Burstein R, Burstein R, Koffler H, et al. Incidence and evolution of subependymal and intraventricular hemorrhage: a study of infants with birth weights less than 1,500 gm. J Pediatr 1978;92:529–34. https://doi.org/10.1016/s0022-3476(78)80282-0.

[50] Schmölzer GM, Reilly MO, Fray C, Os S van, Cheung P-Y. Chest compression during sustained inflation versus 3:1 chest compression:ventilation ratio during neonatal cardiopulmonary resuscitation: a randomised feasibility trial. Archives Dis Child - Fetal Neonatal Ed 2018;103:F455. https://doi.org/10.1136/archdischild-2017-313037.

[51] Schmölzer GM, Pichler G, Solevåg A, Fray C, Os S van, Cheung P-Y. The SURV1VE trial—sustained inflation and chest compression versus 3:1 chest compression-to-ventilation ratio during cardiopulmonary resuscitation of asphyxiated newborns: study protocol for a cluster randomized controlled trial 2019:1–10. https://doi.org/10.1186/s13063-019-3240-8.

[52] Pichler G, Urlesberger B, Baik-Schneditz N, Schwaberger B, Binder-Heschl C, Avian A, et al. Cerebral Oxygen Saturation to Guide Oxygen Delivery in Preterm Neonates for the Immediate Transition after Birth: A 2-Center Randomized Controlled Pilot Feasibility Trial. J Pediatr 2016;170:73-8.e1-4. https://doi.org/10.1016/j.jpeds.2015.11.053.

[53] Ngan AY, Cheung P-Y, Hudson-Mason A, O’Reilly M, Os S van, Kumar M, et al. Using exhaled CO2 to guide initial respiratory support at birth: a randomised controlled trial. Archives of Disease in Childhood-Fetal … 2017;102:F525–31. https://doi.org/10.1136/archdischild-2016-312286.

[54] Kirpalani HM, Ratcliffe SJ, Keszler M, Davis PG, Foglia EE, Pas AB te, et al. Effect of Sustained Inflations vs Intermittent Positive Pressure Ventilation on Bronchopulmonary Dysplasia or Death Among Extremely Preterm Infants. JAMA : The Journal of the American Medical Association 2019;321:1165–11. https://doi.org/10.1001/jama.2019.1660.

# 
